# Moxibustion for the treatment of primary dysmenorrhea: Protocol for an overview of systematic reviews

**DOI:** 10.1097/MD.0000000000018908

**Published:** 2020-01-24

**Authors:** Xue Wang, Jun Xiong, Jun Yang, Ting Yuan, Hao Fan, Yunfeng Jiang, Xiaohong Zhou, Kai Liao, Lingling Xu

**Affiliations:** a Jiangxi University of Traditional Chinese Medicine; b Affiliated Hospital of Jiangxi University of Traditional Chinese Medicine, Nanchang, P.R. China.

**Keywords:** moxibustion, overview, primary dysmenorrhea, systematic reviews

## Abstract

**Background:**

Primary dysmenorrhea (PD) is a common gynecological disease, it refers to spasmodic pain in the lower abdomen before, after or during menstruation, accompanied by general discomfort, In severe cases, fainting may occur due to severe pain, reducing the quality of patients’ life and imposing a heavy burden on social medical security system. There are many ways to treat primary dysmenorrhea, including western medicine and traditional Chinese medicine. Moxibustion is one of the traditional Chinese medicine treatments for primary dysmenorrhea, especially popular in China. Therefore, our overview aims at evaluating the methodological bias and the reliability of the conclusions of systematic reviews (SRs) about moxibustion for primary dysmenorrhoea, and help clinical decision makers translate this research into clinical policy and practice.

**Methods:**

We will search electronic databases including PubMed, Embase, Cochrane Library, Chinese Biomedical Literatures Database (CBM), China National Knowledge Infrastructure (CNKI), WangFang Database (WF), Chinese Scientific Journal Database (VIP) from inception to February 2017. We will consider systematic reviews and meta-analysis of randomized controlled trials evaluating the effect of moxibustion for PD. Two reviewers will identify relevant studies, extract data information, and then assess the methodological quality by Assessment of Multiple Systematic Reviews-2 (AMSTAR-2) tool. Using the Preferred Reporting Items for Systematic Reviews and Meta-Analyses (PRISMA) report checklist to assess the quality of reports included in the study. We will use the evaluations of the Classification of Recommendations, Evaluation, Development and Evaluation (GRADE) of the authors of the included systematic reviews. The screening of systematic reviews, eligibility evaluation, data extraction, methodological quality, and quality of evidence will be conducted by independent reviewers in pairs. The outcomes of interest include: total effective rate, visual analog scale scores (VAS), Cox Menstrual Symptom Scale (CMSS), Dysmenorrhea symptom score and adverse events outcomes prioritized in the individual reviews. We will extract data onto a predefined form designed to summarize the key characteristics of each review. The evidence will be a narrative synthesis organized around the type and content of the intervention and the results reported.

**Results:**

The results of this study will be published in a peer-reviewed journal.

**Conclusions:**

We expect to compile evidence from multiple systematic reviews of symptomatic improvement in patients with primary dysmenorrhea in an accessible and useful document.

**Registration number PROSPERO:**

CRD42019141130

## 1 Introduction

Primary dysmenorrhea (PD), also known as functional dysmenorrhea, refers to the absence of obvious organic lesions in the reproductive system, abdominal pain, low back acid, lower abdominal drop pain, and other discomfort before and after menstruation and during menstruation, which may be accompanied by nausea, vomiting, diarrhea, dizziness, and fatigue.^[1]^ Menstrual disorders are highly prevalent among women, and most commonly feature period pain and mood disturbances. Primary dysmenorrhea (period pain) affects around 3 quarters of all women during their reproductive life, and is especially common in young women in their teens and early adult life.^[2]^ Epidemiological survey shows that the incidence of dysmenorrhea in Chinese women is 33.1%, of which 53.2% are PD, and 13.5% are seriously affected by work and life.^[3]^ A new cross-sectional study shows that the prevalence of PD among Chinese female university students is 41.7%, age at menarche younger than 12 years, irregular menstrual cycle, and skipping breakfast were associated risk factors of primary dysmenorrhea. The prevalence of PD among Chinese university students is relatively high.^[4]^ With the increase of life pressure and the change of diet structure, the incidence of PD is increasing year by year, which has a serious impact on women's life and work.^[5]^

The pathogenesis of PD is complex, and is considered to be the result of multiple factors. The main pathological changes are strong contraction, ischemia and hypoxia of uterine smooth muscle, and spiral artery of uterine wall.^[6,7]^ Many studies have shown that oxidative stress and lipid peroxidation are involved in the development of PD.^[8]^ PD is closely related to the abnormal synthesis and secretion of prostaglandins in endometrium.^[9,10]^ Modern medicine believes that the increase of PGF_2α_ synthesis and release in endometrium during menstruation is one of the main factors of dysmenorrhea. PGF_2α_ acts on the uterus, induces the spasmodic contraction of smooth muscle of uterus, leads to the decrease of blood flow of uterus, and the accumulation of acid metabolites produced by uterine ischemia and hypoxia in the myometrium leads to the occurrence of pain.^[11,12]^

For the treatment of PD, western medicine mainly uses antispasmodic and analgesic drugs, non steroidal anti-inflammatory drugs (NSAIDs), contraceptives, etc. Although it can temporarily relieve the pain, but the recurrence rate is high, and there are different degrees of side effects, easy to produce drug resistance, poor long-term effect, not easy to be accepted by patients.^[13]^ Therefore, patients often select non-pharmacological treatments as an alternative option or as an add-on treatment. According to traditional Chinese medicine theory, dysmenorrhea is mainly caused by mood disorder, stagnation of liver Qi, obstruction of blood flow or contraction of cold and dampness, obstruction of Qi by cold and dampness in uterus, and then weakness of Qi and blood or deficiency of liver and kidney.^[14]^ It is believed that moxibustion with moxa leaves can clear Qi and blood and warm the uterus. By using the heat produced by hot moxibustion and the properties of moxa leaves, the effect of warming yang for dispelling cold, activating blood circulation, and unblocking collaterals can be effectively exerted. Moxibustion is a simple treatment with low economic cost. Because it does not touch the skin, it will not have pain and the patient's acceptance is high.^[15]^ Some related studies have shown that Moxibustion can improve hemorheology, reduce the content of PGF_2α_ in uterine tissue, and improve the activity of NK cells in the spleen of rats.^[16–18]^

Plenty of randomized controlled trials have been conducted to examine the effect of moxibustion treatments on PD,^[19–23]^ and multiple meta-analysis on the basis of randomized controlled trials have therefore been performed.^[24–27]^ Many of the meta-analysis showed that moxibustion treatments have some benefits for patients with PD. However, no critically designed overview to evaluate the systematic review of moxibustion for AR has been carried out so far. Therefore, the purpose of this review is to explore the methodology and reporting quality of existing systematic reviews (SRs), and to summarize the clinical evidence for the treatment of PD by moxibustion, so as to provide patients, doctors, and clinical researchers with information on the current evidence credibility and future research directions.

## 2 Methods

### 2.1 Study registration

It is an overview protocol that follows the recommendations of the Cochrane Handbook for Systematic Reviews of Interventions.^[28]^ This protocol was recorded in the Prospective International Registry of Systematic Review (PROSPERO), registration number CRD42019141130. (http://www.crd.york.ac.uk/PROSPERO/display_record.php?ID=CRD42019141130). And if there are any changes, we will describe it in our full review.

### 2.2 Inclusion and exclusion criteria

Population, Intervention, Comparison, Outcome and Study (PICOS) strategy was employed.

#### 2.2.1 Types of study

For this overview, only systematic reviews and meta-analysis of randomized controlled trials (RCTs) for moxibustion in people with PD, published in English and Chinese, will be included.

#### 2.2.2 Type of participants

Systematic reviews of people are definitely diagnosed with primary dysmenorrhea will be include. Study participants in different age ranges with all types of PD can be included, regardless of sex, race, occupation, education, nationality, etiology, severity.

#### 2.2.3 Type of interventions

Systematic reviews that moxibustion for PD, moxibusition as a single intervention, or major part of a combination therapy with other active intervertion (e.g., conventional drugs, acupuncture, Chinese herbs, etc) will be included.

#### 2.2.4 Type of comparator (s)/control

There is no limit to the treatment of the control group, including no treatment, or placebo, or any control considered for comparison in the individual system review.

#### 2.2.5 Types of outcome measurements

##### 2.2.5.1 Primary outcomes

Primary outcome was the total effective rate of moxibustion in the treatment of primary dysmenorrhea.

##### 2.2.5.2 Secondary outcomes

Secondary outcomes mainly include the following aspects:

1. visual analog scale (VAS) scores.
2. Cox Menstrual Symptom Scale (CMSS).
3. Dysmenorrhea symptom score
4. Advert events.

#### 2.2.6 Study design

SRs containing >1 RCT were included. Non-RCT SRs, review comments, overviews of SRs, editorials, and guidelines were excluded.

### 2.3 Search methods for identification of studies

We searched 3 foreign electronic databases (Cochrane Library, Embase, PubMed) and 4 Chinese electronic databases (China National Knowledge Infrastructure [CNKI], WangFang Database, Chinese Biomedical Literature Database [CBM], and Chinese Scientific Journal Database [VIP]) to collect potential systematic reviews (SRs) from their inceptions to February 2020. The language of publication is limited to Chinese or English. The language within in Chinese and English. The following search terms will be used: dysmenorrhea, functional dysmenorrhea, primary dysmenorrhea, menstrual pain, period pain, moxibustion, thunder fire needle, thunder fire god moxibustion, taiyi miraculous moxa roll, suspended moxibustion, mild moxibustion, needle warming moxibustion, systematic review, meta-analysis, etc. A draft search strategy using PubMed, one of the planned electronic databases to be searched, is presented in Table 1.

**Table 1:**
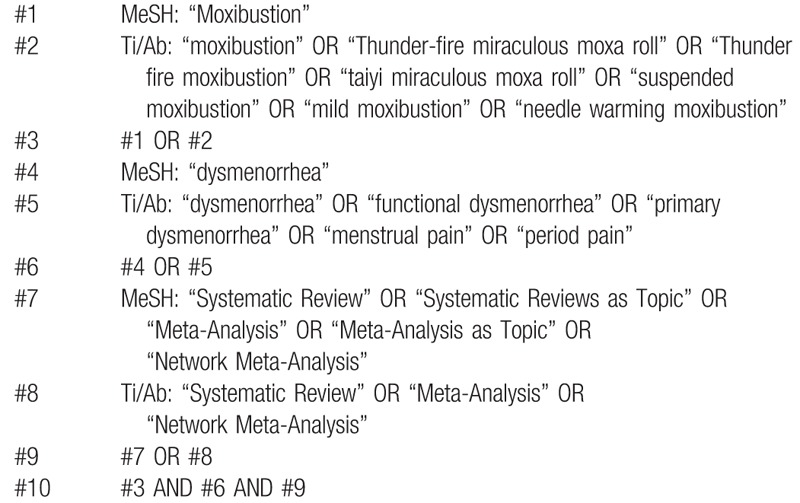
Search strategy (PubMed).

### 2.4 Studies selection

**Figure 1:**
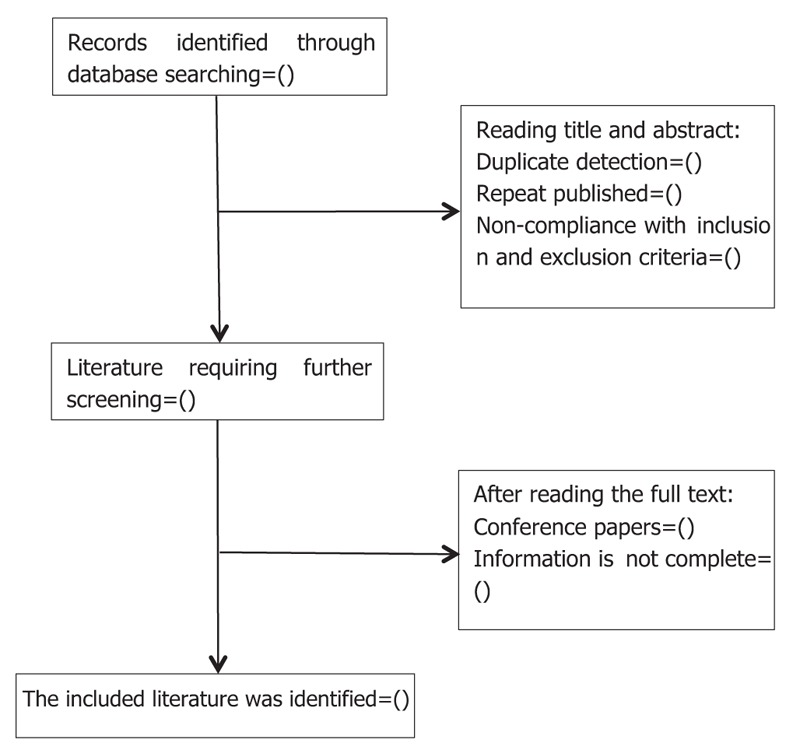
Flowchart of literature selection.

The bibliographies yielded by the literature search will be imported into NoteExpress 3.2.0 for management. Two reviewers (JY and XHZ) will independently read the literature titles, abstracts, and full texts, in sequence, to identify eligible SRs. Any differences will be resolved through discussion to reach a consensus or by using a third author (TY) to adjudicate. The planned selection process is shown in a flow chart (Fig. 1).

### 2.5 Data extraction

Two reviewers (KL and LLX) will independently extract the following data: authors’ name, publication year, country, language, sample size, participants, intervention(s), comparison(s), outcome(s), and some relevant characteristics from the full-text. If the data reported is insufficient or missing, JY will attempt to contact the author for further information to supplement the missing data. In case of any divergence, we will resolve it through discussion and decision by both parties or by consensus with the third reviewer (XW).

### 2.6 Evaluation of the methodological quality of the included studies

Two reviewers (XW and TY) will use the Assessment of Multiple Systematic Reviews-2 (AMSTAR-2) measurement tool to independently evaluate the methodological quality of each systematic evaluation meeting the inclusion criteria.^[29]^ This is most commonly used to assess the quality of systematic reviews included in overviews. AMSTAR-2 is an update of AMSTAR, which can be used to appraise SRs of both randomized and non-randomized controlled trials. AMSTAR-2 includes 16 items, with each of the 16 criteria given a rating of “yes” (definitely done), “no” (definitely not done), “can’t report” (unclear if completed), or “not applicable” based on information provided by the systematic reviews on which reviewers put an evaluation when the criterion is met. The differences will be resolved through discussions between them and, if necessary, arbitrated by a third general author (JY). The results of the methodological quality assessment of the included reviews will be included in an additional Table 2.

**Table 2:**
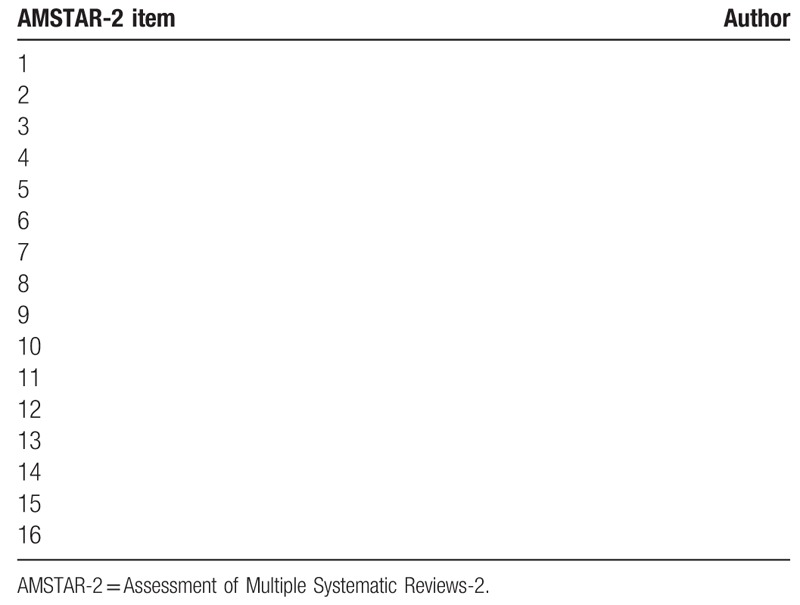
.

### 2.7 Evaluation of the reporting quality of the included studies

Two authors (KL and TY) of the overview will independently evaluate the reporting quality in each review included to assess whether they met the criteria specified in the Preferred Reporting Items for Systematic Reviews and Meta-Analyses (PRISMA).^[30]^ In case of any difference, it will be settled through discussion between them and arbitrated by a third general author (XW) if necessary. The results of the methodological quality assessment included in the review will be included in Table 3.

**Table 3:**
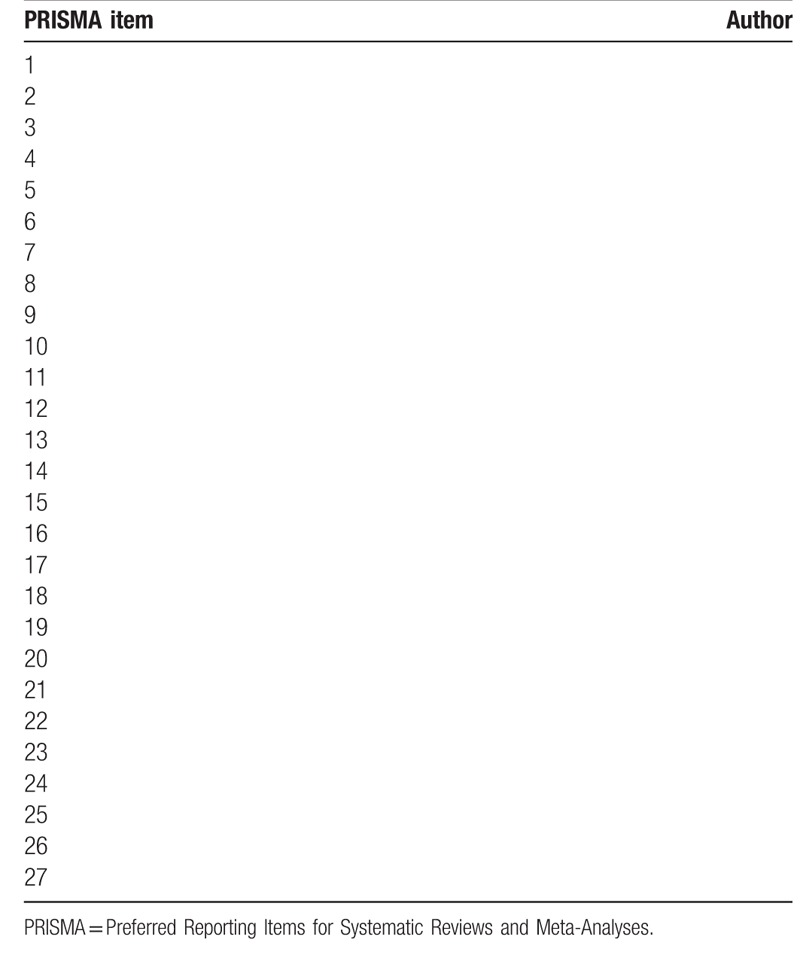
.

### 2.8 Evaluation of the evidence quality of the included studies

The quality of evidence of the included SRs was assessed by the Grading of Recommendations Assessment, Development and Evaluation (GRADE) approach.^[31]^ This tool was designed to evaluate the quality of evidence for each outcome measure across studies. Two authors (HF and JY) will independently assessed the evidence of the outcomes, and the downgraded or upgraded factors affecting the quality of evidence should be described in detail to guarantee the reliability and transparency of results. Any disagreements will be resolved through discussion by 2 authors. The overall quality of evidence was judged as “high,” “moderate,” “low,” or “very low.”

### 2.9 Dealing with lost data

If there is no specific or insufficient data in the published SRs, the author will be contacted by email, or telephone to provide the necessary information. The data will be discarded if we fail to get sufficient data. The analysis will be conducted based on available data, and the potential impact of missing data will be discussed.

### 2.10 Synthesis of data

The analysis unit of this overview is systematic reviews (not individual trials). General characteristics of the eligible trials will be summarized and described, including the total sample size of a meta-analysis, interventions, and their effect size and related 95% CIs. We will provide AMSTAR2 and PRISMA assessments in tabular form for each review to show the risk of bias in the reviews contained in the overall quality and overview, and the total percentage and the 95% CI of each item were calculated. The quality of evidence will be detailed in the form of tables. We combined the reviews in a narrative summary, structured around the type and content of interventions and the reported results.

## 3 Discussion

This study will be an overview of systematic reviews. It will summarize the evidence of moxibustion therapy for PD from a variety of meta-analyses. As far as we know, this will be the first study in this field. In the discussion section in the full report of our study, we plan to include the following subsections, typical for this type of study: summary of main findings; strength and limitations; comparison with other studies and opinions; interpretation of results; and conclusion. The results of this review will provide patients, doctors, and clinical researchers with information about the credibility of current evidence and research direction in the future.

## Author contributions

**Conceptualization:** Xue Wang.

**Data curation:** Jun Yang, Xiaohong Zhou, Ting Yuan.

**Formal analysis:** Kai Liao, Lingling Xu.

**Investigation:** Jun Xiong, Xue Wang, Yunfeng Jiang.

**Methodology:** Xue Wang, Ting Yuan, Jun Yang, Hao Fan.

**Software:** Xiaohong Zhou, Lingling Xu.

**Supervision:** Jun Xiong, Hao Fan.

**Writing – original draft:** Jun Xiong, Xue Wang, Ting Yuan, Jun Yang.

**Writing – review & editing:** Yunfeng Jiang, Xiaohong Zhou, Lingling Xu, Kai Liao, Hao Fan.
